# Different Outcomes in the Acquisition of Residual V2 and *Do*-Support in Three Norwegian-English Bilinguals: Cross-Linguistic Influence, Dominance and Structural Ambiguity

**DOI:** 10.3389/fpsyg.2018.02130

**Published:** 2018-11-09

**Authors:** Merete Anderssen, Kristine Bentzen

**Affiliations:** Department of Language and Culture, UiT – The Arctic University of Norway, Tromsø, Norway

**Keywords:** bilingualism, English, Norwegian, *do-*support, verb second, residual verb second, cross-linguistic influence, language dominance

## Abstract

This paper investigates the acquisition of residual verb second (V2) in three corpora consisting of data from Norwegian-English bilinguals (Emma, Emily and Sunniva) in order to determine to what extent these structures are affected by cross-linguistic influence (CLI) from Norwegian V2. The three girls exhibit three different patterns with regard to the relevant constructions. They are very target-like in their use of auxiliaries in the relevant structures. However, when it comes to *do-*support, Emily and Sunniva are equally target-like, while Emma mainly produces non-target-like structures. These either involve the omission of *do*, or non-target-like movement of a lexical verb. Furthermore, Emma also allows verb movement across the subject with both lexical verbs and auxiliaries in topicalised structures, suggesting that she has overgeneralised residual V2 across verb types and clause types. Emily, on the other hand, is very target-like in structures involving residual V2 in English, but also allows auxiliaries and dummy-*do* to move across the subject in topicalised structures, overgeneralising residual V2 to apply to non-subject-initial declaratives. Finally, Sunniva is very precocious and very target-like in all the relevant structures, which may be an indication of acceleration due to CLI from Norwegian V2. We discuss these results with reference to language balance, finding that the measures available to us suggest that the differences between the children cannot straightforwardly be explained by language dominance. Instead, we suggest that these results can be accounted for by ambiguity in the English system, leaving the data open to several possible interpretations when acquired in contact with the consistent V2 system in Norwegian. This has several consequences: (i) the three girls’ parsers interpret the input differently, (ii) differences between the three children are qualitative rather than quantitative and (iii) there has to be some mechanism that ensures that the children can ‘recover’ from these non-target-like grammars. In this paper, we will focus on the first two issues.

## Introduction

While it is generally agreed that bilingual children separate their two languages from very early on (cf. De Houwer, 2009 for an overview), it is also clear that the two languages of bilingual children may influence each other. Cross-linguistic influence (CLI) is indeed a typical characteristic of bilingual first language acquisition (cf. Serratrice, 2013 for an overview). CLI may have several potential consequences, the most common one being a delay in the acquisition of a particular feature (e.g. Sorace, 2005; Patuto et al., 2011). However, CLI has also been shown to result in a developmental path for bilinguals that *diverges* from that found in monolinguals (e.g. Anderssen and Bentzen, 2013) and in some cases also leading to accelerated development, sometimes in combination with language dominance (e.g. in Kupisch and Bernardini, 2007) but not always (Liceras et al., 2011).

Concerning the underlying causes for CLI, various sources have been explored. Hulk and Müller (2000) and Müller and Hulk (2001) proposed that the syntax-pragmatics interface was particularly vulnerable to CLI. This proposal has been further developed in work by among others Serratrice et al. (2004, 2009), Sorace et al. (2009) and Liceras et al. (2011). Moreover, Hulk and Müller also proposed that CLI would be more plausible in contexts where the two languages display superficial structural overlap. This may lead the child to pose parallel structural analyses to a certain construction in the two languages even in cases where the two languages actually are underlyingly different. Finally, language dominance is another factor that has been explored as a cause for CLI, in particular in explaining the *direction* of CLI (e.g. Genesee et al., 1995; Bernardini and Schlyter, 2004; Foroodi-Nejad and Paradis, 2009).

In the current paper, we address the cause and nature of CLI by investigating the acquisition of *do-*support and residual V2 in three English-Norwegian bilingual children. There is substantial superficial structural overlap with respect to word order between English and Norwegian, as both languages have a basic SVO word order. However, Norwegian is a V2 language, and as a result, *all* finite verbs consistently move to the second position in matrix clauses. English, on the other hand, is not a V2 language but nevertheless has a number of structures exhibiting V2-like characteristics, and it is often referred to as residual V2 (Rizzi, 1996).

The relevant contexts we investigate in this paper are illustrated in (1)–(5). English has V-to-T movement with auxiliaries and *be*, which is visible in clauses with negation or adverbials (1). Moreover, in interrogative clauses, English displays Subject Auxiliary Inversion (SAI) (2). However, in both negative and interrogative clauses, *do*-support is required in the absence of a finite auxiliary [(3), (4)]. In non-subject-initial declaratives, so-called topicalised constructions, English does not display SAI (5).

(1) I *have* not seen her.
(2) *Have* you seen her?
(3) I *did* not buy the book.
(4) *Did* you buy the book?
(5) (a) Today I *have* bought a new car.
      (b) Yesterday I watched the new Star Wars movie.

The three bilingual girls in this study display three different patterns with respect to the acquisition of residual V2, *do*-support and non-subject-initial clauses in English that all diverge from what is typically found in monolingual English-speaking children. We will therefore explore whether all these three outcomes of the bilingual situation are due to CLI from Norwegian V2.

The paper is structured as follows. In the ‘Background’ section, we first provide the relevant background on target-like *do*-support and verb placement in English and Norwegian. We highlight where the two languages display superficially overlapping surface structures, and where the two systems are underlyingly (and superficially) different, thus pointing out where CLI due to structural overlap might be expected. In the ‘Previous Research on the Acquisition of Auxiliaries and *Do*-Support in English’ section, we present previous research on *do*-support and verb placement in monolingual English-speaking children, as well as previous research on the acquisition of verb placement in children acquiring English alongside a (Germanic) V2 language. Finally, in the ‘Research Questions and Predictions for the Current Study’ section, we present the research questions of the current paper. In the ‘Materials and Methods’ section, we introduce the three bilingual girls studied in this investigation, as well as our methodology. In the ‘Results’ section, we present the results of our investigation. In the ‘Discussion’ section contains a discussion of the results, and we explore to what extent the differences between the three girls can be attributed to language dominance. In the ‘Conclusion’ section concludes the paper.

## Background

### Verb Placement in Norwegian and English

In this section, we outline the crucial background on verb placement in Norwegian and English, highlighting areas of superficial structural overlap that might be susceptible to CLI.

#### Verb Second in Norwegian

Norwegian is an SVO language, and as a result, the verb will generally precede the object (6).

(6) Jeg *så* bilen.
      *I saw car.the*
      ‘I saw the car’.

Furthermore, like its Germanic relatives (except English), it is also a V2 language. This means that the finite verbal element moves to the second position in all main clauses (typically analysed as verb movement to the CP domain, cf., e.g. Vikner, 1995). In this position, both finite auxiliaries and finite lexical verbs will precede not just the object, but also negation and other adverbs (7).

(7) (a) Jeg *har ikke* sett bilen.
           *I have not seen car.the*
           ‘I haven’t seen the car’.
      (b) Jeg *så ikke* bilen.
           *I saw not car.the*
           ‘I didn’t see the car’.

Moreover, interrogatives, illustrated by *yes/no-*questions in (8a,b) and *wh-*questions (8c,d), as well as in topicalised structures (9), V2 leads to inversion of the finite verb and the subject.^1^

(8) (a) *Har du* sett den?
           *Have you seen it*
           ‘Have you seen it?’
     (b) *Så du* den?
           *Saw you it*
           ‘Did you see it?’
     (c) Hva *har du* kjøpt?
           *What have you bought*
           ‘What have you bought?’
     (d) Hva *kjøpte du*?
           *What bought you*
           ‘What did you buy?’
(9) (a) Idag *har jeg* kjøpt en bil.
           *Today have I bought a car*
           ‘Today I have bought a car’.
      (b) Idag *kjøpte jeg* en bil.
           *Today bought I a car*
           ‘Today I bought a car’.

#### Residual Verb Second and *Do*-Support in English

Like Norwegian, English is an SVO language, as illustrated in (10).

(10) I *saw* the car.

In contrast, however, English is not a V2 language but exhibits residual V2. This is a reflection of the fact that modern day English has remnants of a grammatical system that used to be more like the one observed in other Germanic languages today, where the finite verbal element typically was the second constituent in the clause. V2 in modern English is residual in two ways. While the other Germanic languages exhibit V2 in all clause types [cf. (6)–(9) above], V2 only applies in certain clause types in English. Moreover, while any finite verb has to move to the second position in Norwegian, V2 only applies to a subset of verbs in English, viz. auxiliaries. Consequently, only finite *auxiliaries* will precede negation and adverbs, as in (11a). In the absence of a finite auxiliary, the phenomenon of *do*-support emerges in negative declaratives.

(11) (a) I *have not* seen the car.
        (b) I *did not* see the car.

Moreover, in *yes/no-*questions (12a,b) and *wh-*questions (12c,d), residual V2 leads to inversion of the finite auxiliary and the subject (12a,c). Again, in clauses without a finite auxiliary, *do*-support is required (12b,d):

(12) (a) *Have you* seen it?
        (b) *Did you* see it?
        (c) What *have you* bought?
        (d) What *did you* buy?

Finally, in topicalised structures, neither finite main verbs nor finite auxiliaries undergo movement across the subject in English. Furthermore, there is no requirement for *do*-support in the second position in these contexts:

(13) (a) Today *I have* bought a car.
        (b) Today *I bought* a car.

Note, however, that remnants of V2 can be found in certain topicalised structures, for example, clauses introduced by short adverbials such as *here* and *there*.^2^

Crucially, when such structures contain full DP subjects they trigger V2-like structures (14a, 15a), while with pronominal DP subjects they occur without V2 (14b, 15b).

(14) (a) Here *comes the bride*.
        (b) Here *she comes*.

(15) (a) There*’s Noddy*.
        (b) There *he is*.

Even though these structures are infrequent in English, they are relevant in this context because they provide evidence to the learner of a V2 grammar in English.

#### Superficial Structural Overlap Between English and Norwegian and CLI

As mentioned in the ‘Introduction’ section, it has been argued that areas where the two languages in a bilingual situation display superficial structural overlap are particularly vulnerable to CLI. When looking at word order and verb placement in particular in English and Norwegian, there are several similarities. Both languages are SVO (16). Moreover, in negative declaratives, in *yes/no*-questions and in *wh*-questions the two languages display finite auxiliaries in parallel positions (17).

(16) Norwegian: Jeg *så* bilen.
        English: I *saw* the car

(17) (a) Norwegian: Jeg *har ikke* sett bilen.
             English: I *have not* seen the car
        (b) Norwegian: *Har* du sett den?
             English: *have* you seen it?
        (c) Norwegian: Hva *har du* kjøpt?
             English: What *have you* bought?

In addition, English has verb movement of finite auxiliaries across the full DP subject in clauses introduced by adverbials such as *here/there*. This yields the same word order as in Norwegian:

(18) Norwegian: Her *kommer bruden*.
        English: Here *comes the bride*.

However, when the finite verbal element is a lexical verb, rather than an auxiliary, the overlap breaks down. In Norwegian, lexical verbs also move to the second position in negative and interrogative clauses, while English employs *do*-support in these contexts (19).

(19) (a) Norwegian: Jeg *så ikke* bilen.
             English: *^∗^I saw not the car*
             I *did not* see the car
       (b) Norwegian: *Så du* den?
             English: *^∗^Saw you it?*
             *Did you* see it?
       (c) Norwegian: Hva *kjøpte du*?
             English: *^∗^What bought you?*
             What *did you buy*?

In addition, the two languages show distinct patterns in non-subject-initial declaratives, where again, Norwegian has a consistent V2 pattern, while English has no verb movement to the second position (20):

(20) (a) Norwegian: Idag *har jeg* kjøpt en bil.
             *English: ^∗^Today have I bought a car.*
             English: Today *I have* bought a car.
       (b) Norwegian: Idag *kjøpte jeg* en bil.
             *English: ^∗^Today bought I a car.*
             English: Today *I bought* a car.

We will argue that the superficial structural overlap shown above may lead to CLI.

### Previous Research on the Acquisition of Auxiliaries and *Do*-Support in English

#### Monolingual Children

In this section, we first briefly address previous research on the acquisition of auxiliaries and *do*-support in English focusing on negative declaratives and interrogatives. Then we review some studies on verb placement in bilingual children acquiring English as one of their languages.

It is well known that children go through an early stage in which they systematically omit functional elements marking tense and agreement. However, finite verbs are rarely completely absent from child grammars at this stage [commonly referred to as the Optional Infinitive (OI) stage, see, e.g. Harris and Wexler, 1996]. According to de Villiers and de Villiers (1985), auxiliaries enter English when children reach the two–three word stage. However, all auxiliaries do not come in simultaneously. Stromswold’s (1990) extensive corpora study of 12 children (age range 1;2–7;10) shows that the first functional verbal element to appear is copula *be*, which on average is first attested at 2;2 in her data. A couple of months later, at 2;7, the first use of auxiliary *be* is found. The age of the first use of *do*-support is on average 2;8, while auxiliary *have* is the last, and only attested in Stromswold’s data as late as at 3;5. See also Rispoli et al. (2012) for similar findings for copula *be*, *do* and auxiliary *be*. They do not discuss auxiliary *have*.

Most of the earlier studies on the acquisition of auxiliaries in negated and interrogative clauses have focused on auxiliaries other than *do*. One notable exception is Ervin-Tripp (1973) and Miller (1973), who describe *do-*support as typically first attested in negative declaratives, and subsequently expanded to questions. For four of the five children investigated in these studies, the productive use of *do* in negation preceded the use of *do* in questions by 2–7 months. The exception is Susan, who productively employs *do*-support in questions 2 months earlier than in negative declaratives. First attestations of *do* in both questions and negative declaratives is at age 2;2 for Susan. Fletcher’s (1985) case study of Sophie finds a similar asymmetry where *do-*support is used in declaratives clauses prior to questions.

For negative structures, the developmental path has been argued to involve an initial stage of pre-sentential negation, such as *No the sun shining* (Déprez and Pierce, 1993: 34, see also Bellugi, 1967 for an early description of this). These types of negative declaratives may occur with either *no* or *not*, and with or without the subject present. However, Drozd (1995) shows that only 10 of the 123 children investigated in his study produced at least one such structure, suggesting that not all children exhibit this behaviour. At the next developmental stage, children tend to produce structures with sentence medial *no* or *not* where the obligatory auxiliary typically is omitted, such as *Man no go in there* and *Wayne not eating it* (Radford, 1994: 152, 153). Radford refers to this as the (pre-functional) lexical-thematic stage, due to the fact that most main clauses are non-finite, most typically in the infinitive form. According to the original study in Bellugi (1967), children start using the negative forms *can’t* and *don’t* at this stage, but these represent unanalysed chunks, as auxiliaries generally tend to be absent. The frequent occurrence of non-agreeing *don’t* has been related to the absence of adultlike tense and agreement at the OI stage (see, e.g. Schütze, 2010; Miller, 2013). At the final developmental stage, children rapidly start making use of auxiliaries in both negative and declarative contexts. This occurs at age 3;2 for Adam and 3;8 for Sarah, while Eve, who is widely considered to be very precocious, reaches this stage at age 2;2. Generally, these studies have not addressed whether there is a difference between the acquisition of *do* and other auxiliaries. However, Rowland and Theakston (2009) report a lower proportion of target-like structures with *do*, compared to other auxiliaries, suggesting that *do* might be more difficult to acquire than auxiliaries in general. This is also true for the younger children in the study in Santelmann et al. (2002). In a recent study, Thornton and Rombough (2015) investigated the acquisition of *do*-support in negative declaratives in 25 children aged 2;5–3;4. They elicited negations where a target-like construction would include auxiliary *doesn’t*. Their results show that more than half of the children’s responses (52.5%^3^) are target-like and include *doesn’t* (their Table 3). Only 10% of the responses contain just a bare main verb (*It not fit*). The most common non-target-like pattern involved non-target-like marking of third person singular (*It’s not fit*, *It not fits*, *It doesn’t fits*). However, the 25 children clearly split into two groups, one advanced group (12 children) and one less advanced group (13 children). The advanced group was target-like (using *doesn’t*) 79% of the time, while the less advanced group only used target-like *doesn’t* 1.4% of the time. In fact, nine of the 13 children in this latter group did not produce any instances of *doesn’t* at all. The most common errors in this group involved either the pattern *It not V(s)* (33.1%) or non-agreeing *don’t* [*It don’t V(s)*] (17.2%). Notably, with respect to age, there does not seem to be any significant differences; both groups contain children within the whole age range from 2;5 to 3;4. This suggests that there is a lot of variation concerning at what age productive *do*-support in negative declaratives is acquired.

Turning to interrogatives, several studies have shown that auxiliaries tend to be omitted in *wh*-questions at an early stage (Roeper and Rohrbacher, 1994; Bromberg and Wexler, 1995). In a study on the acquisition of finiteness in English (and Norwegian) *wh*-questions, Westergaard and Bentzen (2010) investigate data from seven English-speaking children [Adam (3;0–3;5) and Sarah (2;9–5;1) from the Brown corpus, Brown, 1973; MacWhinney, 2000, and five children from the Manchester corpus, Warren, Anne, Ruth, Liz and Nicole ranging from 1;10–3;0, Theakston et al., 2001]. They report that copula *be* is much less frequently omitted compared to auxiliaries. Moreover, dummy-*do* and auxiliary *be* are missing much more often than modal auxiliaries. However, Westergaard and Bentzen (2010) do not find a clear distinction between the rate of dummy-*do* and auxiliary *be* omissions. Rather, there seems to be individual variation between the children with respect to which of the two auxiliary types are more frequently missing in *wh*-questions. Finally, their study also shows that *do* and auxiliary *be* are both still omitted quite frequently (for some children more than 50% of the time) up to the age of at least 2;9. In somewhat contrast to this, Erreich (1984) investigating 18 children aged 2;5–3;0 finds that auxiliaries are present in obligatory contexts in *wh*-questions and *yes/no*-questions (as well as declaratives) more than 80% of the time.

Concerning interrogatives, when auxiliaries are present in children’s questions, SAI is typically employed. While some studies have reported that young children sometimes produce interrogatives without inversion (e.g. Klima and Bellugi, 1966 for *wh*-questions, Erreich, 1984), Santelmann et al. (2002) point out that few studies have been able to show a stage that completely lacks SAI. Comparisons of the rate of SAI in *yes/no*-questions and *wh*-questions show variable results. Some studies do not find differences between the two types of interrogative clauses (e.g. Stromswold, 1990), others report that children more accurately and frequently make use of SAI in *yes/no*-questions than in *wh*-questions (Klima and Bellugi, 1966; Bellugi, 1971; Rowland, 2007; Pozzan and Valian, 2017), while yet others argue that SAI is employed earlier or more consistently in *wh*-questions than in *yes/no*-questions (Erreich, 1984; Valian et al., 1992).

Summing up, between ages 2 and 3 monolingual English-speaking children do not consistently include auxiliaries in negated and interrogative clauses, although inclusion of such elements gradually becomes the dominating pattern. The inclusion of dummy-*do* does not clearly lag behind the acquisition of other auxiliaries. Moreover, once auxiliaries are overtly expressed in negated and interrogative clauses, the typical patterns are Aux-Neg and SAI, although lack of SAI does occur in questions. Notably, to our knowledge, no studies report on non-target-like verb movement in monolingual English first language acquisition.

#### Bilingual Children

The children in our study are acquiring English alongside the V2 language Norwegian, and we explore the effect this might have on the acquisition of verb placement in English. Although this has not been investigated for English/Norwegian bilingual children previously (though see Bentzen, 2000 for a preliminary study of one of the children in the current investigation), a few studies have looked at children acquiring English alongside other V2 languages. In an extensive case study, Knipschild (2007) investigates the acquisition of verb placement in the German/English bilingual boy Joshua, from age 2;4–3;1. While he appears to have acquired target-like V2 in German early on, he displays non-target-like behaviour in English. More specifically, he (predominantly at the earliest stages) produces structures that suggest verb movement of a lexical verb in negated and interrogative clauses (21), (22). He also employs verb movement in non-subject-initial declaratives (23). Furthermore, *do*-support only comes in after the age of 2;9, and is initially often used in non-target-like manners, e.g. uninverted (24a) or in declaratives as a superfluous *do* in non-emphatic contexts (24b) (from Knipschild, 2007: 92, 136):

(21) I *want* not some water. (Joshua 2;4)
(22) What *make* the kittens? (Joshua 2;10)
(23) The flower *throw* daddy in the water. (Joshua 2;9)
(24) (a) Where I *did* get this from? (Joshua 2;10)
        (b) I *did* watch it. (Joshua 2;10)

In fact, more than 90% of negated clauses and *wh*-questions displayed the patterns in (21) and (22) in the early stage (age 2;4–2;9). As pointed out in the previous section, monolingual English-speaking children hardly ever produce this kind of verb movement. Knipschild argues that the non-target-like utterances in (21)–(23) above are due to transfer from German. Similar findings in the English of bilingual German/English children have been reported by Döpke (1998, 1999), Schelletter (2000) and Genske (2014).

In a case study of an Icelandic/English bilingual girl Katla, Bohnacker (2013) reports that while the child does use *do*-support, this is only employed in negative declaratives between the ages of 2;0 and 2;11 and in questions from the age of 3;0.

### Research Questions and Predictions for the Current Study

As highlighted in the ‘Verb Placement in Norwegian and English’ section, there is considerable superficial structural overlap between Norwegian and English, suggesting that CLI can be expected. Moreover, studies of bilingual children acquiring English together with other Germanic V2 languages reveal that such influence does occur. Given this, our research questions are as outlined in (1)–(3), and we make the predictions in (4) and (5).

(1) To what extent is the acquisition of residual V2 and *do-*support in English affected by simultaneous acquisition of Norwegian?
  (a) Is residual V2 expanded to apply to *all verb types*, including lexical verbs?
  (b) Is residual V2 expanded to apply to *all clause types*, including topicalised structures?
  (c) Is residual V2 expanded to both *all verb types* and *all clause types*, resulting in a full V2 system?
  (d) Is the acquisition of residual V2 and especially *do-*support delayed or accelerated?

(2) Are all the three children affected by CLI from Norwegian in the same way?
(3) If they are not, what can explain the differences?
(4) If CLI from V2 in Norwegian affects the acquisition of residual V2 and *do-*support in bilingual children, the following logical possibilities exist:
  (a) If residual V2 is expanded to apply *to all verb types*, including lexical verbs:
    (i) the acquisition of *do-*support should be delayed, as it makes the phenomenon superfluous in the grammar, and
    (ii) lexical verbs should occur in the position normally reserved for auxiliaries in questions and negative declaratives.
  (b) If residual V2 is expanded to apply *to all clause types*, SAI and *do-*support should also occur in topicalised structures.
  (c) If residual V2 is expanded to apply *to all verb types and all clause types*, the children should allow a full V2 grammar.
  (d) If CLI from Norwegian V2 accelerates the acquisition of residual V2 and *do-*support, these phenomena should be attested at an earlier stage in bilingual children.
(5) Given that there is a great deal of ambiguity in the English system, it should be possible for different parsers to be affected by simultaneous input from Norwegian V2 in different ways, resulting in different grammars.
(6) If such differences occur, they can be explained as an effect of language dominance.

## Materials and Methods

The current study is a corpus study based on data from three girls, all bilingual from birth: Emma, Sunniva and Emily. These corpora were collected by the authors in connection with previous projects.^4^

As mentioned, the three girls grew up in very similar language situations; they all have one native English-speaking parent and one native Norwegian-speaking parent and grew up in Tromsø, Norway. Thus, English is a heritage language and Norwegian is the majority language in the lives of these children. The Norwegian-speaking parents opted to speak English with their children as well as with their English-speaking spouses. Thus, in all three cases, English is the home language. All girls attended nursery from around the age of one, Emily slightly later as she was born in the summer and started after the summer holiday, at approximately 14 months. Thus, this is the age at which consistent exposure to Norwegian started, even though both families were in close contact with family, friends and society at large, making some exposure to Norwegian likely most days even before the age of 1.

Two of the children, Emma and Sunniva, were also the first child in the family, while Emily has two older siblings, one of them being Sunniva. Emma’s English-speaking parent is her American mother, while Sunniva and Emily’s father is British. Sunniva and Emily also speak English with one another and with their brother. There is a 10-year age difference between the two sisters.

The data from Emma, Sunniva and Emily form the basis of the current study. Relevant information about the three corpora is summarised in Table 1. As Table 1 shows, the corpora are quite spread out in terms of measures such as age, number of files and utterances, and Mean Length per Utterance for Words (MLU_W_). Emma was recorded biweekly in both English and Norwegian in the course of a three-month period between the ages of 2;7.10 and 2;10.9. There are six English files in Emma’s corpus, consisting of 1831 child utterances. In these files, her MLU_W_ range is 3.074–3.998. Sunniva was recorded in English and Norwegian for approximately a year, from age 1;6.25–2;8.0, at irregular intervals. There are nine files and 2512 utterances in her English data. Her MLU_W_ ranges from 1.992–3.667 in these files. The equivalent information about Emma’s and Sunniva’s Norwegian files can be found in Table 1 for comparison. Emily, on the other hand, was only recorded in English, and there are only four files in her corpus. The two first recordings were made just a few days apart, at ages 2;3.19 and 2;3.25, while recordings three and four were made considerably later and approximately a month apart, at 3;8.18 and 3;9.25. Her corpus consists of 1495 child utterances and the MLU_W_ range is 2.833–4.961, with the first two recordings clustering between 2.8 and 3 and the last two ranging from 4.7 to almost 5.

**Table 1:** Overview of the data used in the study.

	Sunniva	Emma	Emily	Total
Age range	1;6.25–2;8.0	2;7.10–2;10.9	2;3.19–3;9.25	1;6.25–3;9.25
Number of English files	9	6	4	19
Utterances in English files	2512	1831	1495	5838
MLU range English files	1.992–3.667	3.074–3.998	2.833–4.961	1.992–4.961
Number of Norwegian files	7	7	Not applicable	14
Utterances in Norwegian files	2890	2222	Not applicable	5112
MLU range Norwegian files	1.932–3.442	3.282–4.120	Not applicable	1.932–4.120


Emma’s files were originally transcribed by the Norwegian investigator and were later checked by a native speaker of English. Sunniva’s and Emily’s files were transcribed by a native speaker of English and subsequently checked by a native speaker of Norwegian. For the current study, the files from the three children were searched manually for the relevant structures. In the searches, all the contexts that obligatorily involve *do-*support or another auxiliary were identified: (i) negative declaratives, (ii) *yes/no*-questions and (iii) *wh-*questions. We included (iv) non-subject-initial declaratives in the searches, some of which also involve auxiliaries or *do-*support (see ‘Residual Verb Second and *Do*-Support in English’ and ‘Superficial Structural Overlap Between English and Norwegian and CLI’ sections).

Direct repetitions, both of other interlocutors and self-repetitions, were generally excluded. For example, the two examples in (25) were only counted as one *wh-*question. However, there are some exceptions to this. First, repetitions were included when the child kept repeating the same sentence but produced it in different forms [e.g. (26) below]. Similarly, when the child repeated an adult utterance incorrectly, the relevant example would be included in the count, but not when the child only repeated a part of the utterance (27).

(25) Sunniva: Where’s the pillow? (1;6.25)
        *Where’s the pillow?*
        Mother: Where’s the pillow?
        Mummy can’t see the pillow from here.
(26) Emily: Where are the cats gone? (3;9.25)
        Where the cats gone?
        *Where the cats gone?*
        Mummy?
        Mother: Hmm?
        Emily: You have to say: Where are the cats gone?
        Mother: Where have the cats gone?
        Emily: Here.
(27) Sunniva: What do you got? (2;1.16)
        Mother: It’s a little mouse, I think.
        *Sunniva: Little mouse, I think*.

Finally, identical repetitions produced by the child because she was explicitly asked to do so by the adult interlocutor were also included in the count. Other structures that were excluded from the counts were utterances that were questioned in the transcription or for which alternative transcriptions were proposed, both indicating that the transcriber was unsure about the relevant utterance. Similarly, when a central part of the utterance is incomprehensible, the relevant example was not included in the count [see, e.g. (28)].

(28) Mother: Mummy gonna put the (.) knickers on the little dolly. (1; 11.22)
        Mother: On this little dolly.
        Mother: I think they go on like this.
        *Sunniva: Where [?] xxx the knickers gone?*
        Mother: Huh?

In other situations, examples where the incomprehensible part did not have any consequences for the relevant phenomena were included. Structures involving utterances where the transcriber was unsure about the transcription or where central parts of it were incomprehensible were double checked with the sound files and included or excluded depending on whether the authors agreed, disagreed or were still unsure about the relevant utterances.

## Results

As Table 2 shows, all three children productively use finite auxiliaries, modals and copula, and include these elements in negative declaratives and interrogatives quite consistently. Relevant examples are provided in (29)–(31). For Emily, non-target-like structures all lack an auxiliary [e.g. (32)], while Sunniva and Emma also have a couple of examples where the auxiliary is present but uninverted in questions [cf. (33)]^5^.

(29) *Can you* sit on my back? (Emily 3;8.18)
(30) Winnie the Pooh*’s not* broken mummy. (Sunniva, 2;6.1)
(31) What*’s that here* on your watch? (Emma 2;8.17)
(32) I *not* doing it. (Emma 2;9.2)
        TARGET: I’m not doing it.
(33) What *I’m* drawing, mummy? (Sunniva 2;6.1)
        TARGET: What am I drawing?

**Table 2:** Target-like use of finite auxiliaries/copula in questions and negative declaratives versus non-target-like structures with missing auxiliaries or lack of SAI in Emma, Emily and Sunniva’s files.

Child	SAI/Aux-neg	No auxiliary	No SAI in	Total
	(%)	(%)	questions (%)	
Emma	90 (86.5%)	10 (9.6%)	4 (3.8%)	104
Emily	135 (95.1%)	7 (4.9%)	0	142
Sunniva	164 (93.2%)	9 (5.1%)	3 (1.7%)	176
Total	389 (92.2%)	26 (6.2%)	7 (1.7%)	422


For contexts requiring *do-*support, on the other hand, the situation is very different, especially for Emma. While 86.5% of her questions and negative structures include the auxiliary, *do-*support is only employed in 16.7% of structures requiring this. Furthermore, even though the majority of non-target-like utterances simply lack *do* (50%), similarly to what has been observed for monolingual English children, close to a third of Emma’s questions and negative structures displays movement of a lexical verb (33.3%). In comparison, Emily and Sunniva supply *do* at a very similar rate to other auxiliaries, at 92.6 and 91.2%, respectively. These results are summarised in Table 3. Examples of target-like and non-target-like structures are provided in (34)–(39).

(34) Where *did you* make [/] make some waffles? (Sunniva 1;11.22)
(35) Cinderella, *do you* want to see? (Emily 3;9.25)
(36) No, I *don’t* watch mmm dance competition. (Sunniva 1;11.22)
(37) I *hurt not* this knee now. (Emma 2;8.5)
        TARGET: I did not hurt this knee now
(38) I *not know*. (Emma 2;8.17)
        TARGET: I don’t know
(39) *Drive daddy* me to barnehage? (Emma 2;8.5)
        TARGET: Will/did daddy drive me to nursery?

**Table 3:** The total use of *do-*support in residual V2 contexts in Emma, Emily and Sunniva.

Child	*Do-*support	No *do*	V2 with lexical	Total
	(%)	(%)	verbs (%)	
Emma	10 (16.7%)	30 (50%)	20 (33.3%)	60
Emily	63 (92.6%)	4 (5.9%)	1 (1.5%)	68
Sunniva	31 (91.2%)	2 (5.9%)	1 (2.9%)	34
Total	104 (64.2%)	36 (22.2%)	22 (13.6%)	162


Considering these examples in more detail, we see that Emma in general is less target-like than Emily and Sunniva with all the structures involving residual V2. Tables 4–6 provide the distribution of SAI for each of the structures requiring an auxiliary or *do-*support in the three children.

**Table 4:** Finite verb placement in *wh-*questions (requiring SAI or *do*-support) in Emma, Emily and Sunniva’s files.

Child	Auxiliaries/copula (%)			*Do-*support (%)			Total (%)	
			
	SAI	Lacking aux/cop	No SAI	SAI	Lacking *do*	V2/lex. verb	Target	Non-target
Emma	6/10	0/10	4/10	1/1	0/1	0/1	7/11	4/11
(11)	(60)	(0)	(40)	(100)	(0)	(0)	(63.6)	(36.4)
Emily	29/32	3/32	0/32	3/3	0/3	0/3	32/35	3/35
(35)	(90.6)	(9.4)	(0)	(100)	(0)	(0)	(91.4)	(8.6)
Sunniva	101/111	8/111	2/111	7/9	1/9	1/9	108/120	12/120
(120)	(91)	(7.2)	(1.8)	(77.8)	(11.1)	(11.1)	(90)	(10)


**Table 5:** Finite verb placement in *yes/no-*questions (requiring SAI or *do*-support) in Emma, Emily and Sunniva’s files.

Child	Auxiliaries/copula (%)			*Do-*support (%)			Total (%)	
			
	SAI	Lacking aux	No SAI	SAI	Lacking do	V2/lex. verb	Target	Non-target
Emma	47/47	0/47	0/47	4/18	0/18	14/18	51/65	14/65
(65)	(100)	(0)	(0)	(22.2)	(0)	(77.8)	(78.5)	(21.5)
Emily	53/53	0/53	0/53	16/20	4/20	0/20	69/73	4/73
(73)	(100)	(0)	(0)	(80)	(20)	(0)	(94.5)	(5.5)
Sunniva	54/56	1/56	1/56	10/11	1/11	0/11	64/67	3/67
(67)	(96.4)	(1.8)	(1.8)	(90.9)	(9.1)	(0)	(95.5)	(4.5)


**Table 6:** Finite verb placementin negative declaratives (requiring an auxiliary or *do*-support) in Emma, Emily and Sunniva’s files.

	Auxiliaries/copula (%)		*Do-*support (%)			Total (%)	
			
Child	Aux-neg	Lacking aux	*Do*-neg	Lacking *do*	V2/lex. verb	Target	Non-target
Emma	37/47	10/47	5/41	30/41	6/41	42/88	**46/88**
(88)	(78.7)	(21.3)	(12.2)	(73.2)	(14.6)	(47.7)	**(52.3)**
Emily	53/57	4/57	44/45	0/45	1/45	97/102	5/102
(102)	(93)	(7)	(97.8)	(0)	(2.2)	(95.1)	(4.9)
Sunniva	9/9	0/9	14/14	0/14	0/14	23/23	0/23
(23)	(100)	(0)	(100)	(0)	(0)	(100)	(0)


As these tables show, Emma is more target-like in *yes/no-*questions (78.5%) than in *wh*-questions (63.6%) and negative declaratives (47.7%). However, as shown in Table 5, the majority of Emma’s *yes/no*-questions (47/65) involve an auxiliary other than *do*, and these are all target-like. Of the 18 *yes/no*-questions requiring *do*-support, only 22.2% are target-like. All the non-target-like *yes/no-*questions involve movement of the lexical verb. As Table 6 shows, most of Emma’s non-target-like negative structures involve the omission of *do* (73.3%). Only a small proportion exhibit lexical verb movement (14.6%). The two other children are more consistent (and target-like) across the various structures.

Turning to non-subject-initial structures, we see that all three children are very different from one another. As shown in Table 7, Sunniva hardly produces any topicalised structures if we exclude topicalisations with *here/there* [cf. (13), (14) in the ‘Residual Verb Second and *Do*-Support in English’ section]. There are only two relevant examples attested in her corpus, and both of these are target-like. Both Emma and Emily produce topicalisations with verb movement. However, while Emma allows verb movement of both auxiliaries and lexical verbs, Emily only exhibits verb movement of auxiliaries. Furthermore, these two girls make use of verb movement at very different rates. As Table 7 shows, Emma employs inversion in close to 30% of topicalised constructions, while Emily produces SAI at approximately 66%. Some examples are provided in (40)–(43).^6^

(40) And *then did* Belle go up again. (Emily 3;8.19)
(41) *Then you* need to take some shampoo. (Emily 3;8.19)
(42) *Now throw* I it. (Emma 2;8.17)
(43) *Now I* step on the scary woman. (Emma 2;8.17)

**Table 7:** Finite verb placement in topicalised constructions, divided into verb types, in Emma, Emily and Sunniva’s files.

Child	Lexical verbs		Auxiliaries/copula		*Do*-support		Total	
				
	Non-V2	^∗^V2	Non-V2	^∗^V2	Non-V2	^∗^V2	Target non-V2	Non-target V2
Emma	22/28	6/28	25/39	14/39	1/1	0/1	48/68	20/68
(68)	(78.6%)	(21.4%)	(64.1%)	(35.9%)	(100%)	(0%)	(70.6%)	(29.4%)
Emily	16/16	0/16	7/33	26/33	3/27	24/27	26/76	50/76
(76)	(100%)	(0%)	(21.2%)	(78.8%)	(11.1%)	(88.9%)	(34.2%)	(65.8%)
Sunniva	1/1	0/1	1/1	0/1	0/0	0/0	2/2	0/2
(2)	(100%)	(0%)	(100%)	(0%)	(0%)	(0%)	(100%)	(0%)


## Discussion

So far, we have seen that both Emily and Sunniva are quite target-like in their use of auxiliaries in *wh-*questions, *yes/no-*questions and negation. With respect to *yes/no*-questions, Emma is also target-like in her use of auxiliaries, while she is somewhat less consistent in *wh*-questions and negative declaratives. However, when it comes to structures that require *do-*support, Emma is much less target-like that Emily and Sunniva. In this section, we discuss the results in more detail, and address the research questions posed in ‘Research Questions and Predictions for the Current Study’. Recall that the main research questions concerned (1) to what extent the acquisition of residual V2 and *do-*support in Norwegian-English bilinguals is influenced by Norwegian V2, (2) whether all the three children are affected in the same way and (3) if they are affected differently, what can explain the differences.

### Different Outcomes of CLI

In what follows, we consider each of the three children in turn with regard to possible CLI from Norwegian. In doing so, we also consider whether the children are affected by this possible influence in the same way, or whether different parsers could possibly interpret the bilingual input in different ways. Recall that we predicted that CLI might take several forms. It might cause residual V2 to be expanded to include all verb types, which would result in lexical verbs preceding the subject in questions and non-subject-initial clauses and preceding negation in negative declaratives. A by-product of such influence could be that *do-*support is obsolete. A second possible outcome of CLI could be the expansion of residual V2 to all clause types, resulting in consistent SAI in topicalised declaratives. CLI might also affect both verb types and clause types, resulting in (the possibility of) a full V2 grammar in the children’s English. A final possibility is that the simultaneous acquisition of Norwegian V2 might accelerate the acquisition of residual V2 and especially *do-*support. The reasoning behind this prediction is that Norwegian V2 word order may enhance the need for a verbal element in a pre-negation position in negative declaratives and in a pre-subject position in interrogative clauses.

#### Emma – Pattern 1: Transfer of Both Verb Types and Clause Types

As we saw in Table 2, auxiliaries and copula are acquired and occur in the target position in Emma’s data 86.5% of the time (90/104). At the same time, Table 3 shows that Emma is considerably less target-like in structures where *do*-support is required. As shown in Table 6, most non-target-like negative declaratives with a lexical verb are characterised by absence of *do-*support (73.2%). In addition, 14.6% of negative declaratives requiring *do*-support instead displays non-target-like movement of the lexical verb, as illustrated by the example in (44). Moreover, Table 5 shows that as much as 77.8% of Emma’s *yes/no-*questions involve non-target-like verb movement of the lexical verb, see (45) below:

(44) I *hurt not* this knee now. (Emma 2;8)
(45) *Drive daddy* me to *barnehage* (=daycare)? (Emma 2;7)

These examples indicate CLI from Norwegian V2 across verb types in Emma’s data. Another indication of this is demonstrated by Emma’s placement of *gonna* (not included in Tables 3, 6). *Gonna* (going to) is not a lexical verb but patterns with lexical verbs with regard to placement in negative declaratives and questions, as illustrated in (46). However, in Emma’s negative declaratives, *gonna* almost exclusively occurs in front of the negation (19/22, 86.4%) (47):

(46) He’s *not gonna* make it.
(47) Now that *gonna not* sleep more. (Emma 2;8)

Furthermore, there are also indications of CLI from Norwegian V2 across clause types in Emma’s data. As revealed by Table 7, Emma allows verb movement of lexical verbs in non-subject-initial structures. 20/68 (29.4%) topicalisations display verb movement/inversion. Two examples are provided in (48) and (49), illustrating this for a lexical verb (48) and a perfective auxiliary (49).

(48) Now *throw I* it. (Emma 2;8)
(49) Now *have I* ringed Angus. (Emma 2;8)

Thus, Emma meets predictions (4a)–(4c). Due to influence from Norwegian V2, V2 appears to be transferred to apply across verb types and across clause types in Emma’s English, making it almost equivalent to her Norwegian grammar in this respect.

#### Emily – Pattern 2: Transfer of Residual V2 to Non-subject-Initial Clauses

As shown in Table 2, Emily includes auxiliaries and copula in the target position to a large extent in her residual V2 structures. However, unlike Emma, her use of *do*-support is also very target-like. Recall that she supplies auxiliaries in questions and negative structures in the target position at 95.1% (135/142) and *do* in the same structures at 92.6% (65/68). There is only one example of V2 with a lexical verb. Thus, it seems clear that Emily has not expanded residual V2 to apply to lexical verbs [thus not meeting prediction (4a)]. However, as illustrated in Table 7, Emily also exhibits SAI in topicalisations at 34.2% (26/76) and displays *do*-support in such contexts at 31.6% (24/76), as illustrated in (50) and (51), making as much as 65.8% (50/76) of topicalised clauses non-target-like.

(50) And then *was madame Gazelle’s telephone* ringing. (Emily 3;8)
(51) And then *did Candy Cat* come. (Emily 3;8)

Thus, we argue that there is CLI from Norwegian V2 into English also in Emily’s data, causing residual V2 to be expanded across clause types, confirming prediction (4b).

#### Sunniva – Pattern 3: Target-Like – And Early?

Finally, Sunniva is also target-like and consistent with respect to her use of auxiliaries and copula, including such elements 93.2% of the time (164/176), as shown in Table 2. She is also very target-like with *do-*support, which is included in 91.2% of required contexts (31/34) (see Table 3). However, note that there seem to be fewer contexts for *do-*support in Sunniva’s files, compared to the other two, which might be related to the fact that Sunniva is younger than the other two children in most of her files. The general impression of Sunniva’s production is that she is very target-like. She does not employ movement of lexical verbs (except in one instance), nor does she produce any non-target-like topicalisations [but notably she only produces two (non-imitated) non-subject-initial structures] (52).

(52) Maybe *he’s* swimming. (Sunniva 1;9.13)

It would thus appear that Sunniva is not affected by CLI in residual V2 structures, contrary to predictions (4a)–(4c).

This leaves prediction (4d), suggesting that CLI from V2 may cause the acquisition of residual V2 and *do-*support to be accelerated. There are some challenges with respect to this issue. For one thing, the three children investigated in the current study are in relatively different age spans. Recall from Table 1 that the children are recorded both at different age span and for different lengths of time. The distribution of recordings for the three children is presented in Table 8.

**Table 8:** Overview of recordings according to age for Emma, Emily and Sunniva.

Age range	Sunniva	Emma	Emily
1;7–1;10	Sunniva (1;6.25)		
	Sunniva (1;9.13)		
1;11–2;2	Sunniva (1;10.01)		
	Sunniva (1;11.22)		
	Sunniva (2;1.16)		
	Sunniva (2;1.21)		
2;3–2;5	Sunniva (2;4.6)		Emily (2;3.19)
			Emily (2;3.25)
2;6–2;10	Sunniva (2;6.1)	Emma	
	Sunniva (2;8.0)	(2;7.14)	
		Emma (2;8.5)	
		Emma (2;8.17)	
		Emma (2;9.2)	
		Emma (2;9.23)	
		Emma (2;10.8)	
2;11–3;1			
3;2–3;5			
3;6–3;9			Emily (3;8.18)
			Emily (3;9.25)


Furthermore, as discussed in the ‘Previous Research on the Acquisition of Auxiliaries and Do-Support in English’ section, *do-*support has been observed to occur in negation before questions in both monolingual and bilingual children. In our data, there is a total of 10 examples of *do-*support in all of Emma’s files, five in negative structures, four in *yes/no*-questions and one in *wh-*questions. In Emily’s first two files, aged 2;3.25 and 2;4.19, she produces 29 instances of *do-*support, four of which are in questions, which at least does not seem to be late compared to monolinguals. In Sunniva’s files, however, there are 12 instances of *do-*support before the age of two; all but one occur in negative structures. Both types are illustrated in (53) and (54).

(53) No, I *don’t* watch mmm dance competition. (Sunniva 1;11.22)
(54) Where *did you* make [/] make some waffles? (Sunniva 1;11.22)

Even though Sunniva clearly makes use of *do-*support at a very young age, it is difficult to say for sure whether this is (i) early compared to monolinguals and (ii) early compared to Sunniva’s general development.

With regard to the first question, Miller (1973), which is also based on corpus data, shows that Susan’s first example of *do-*support in a negative clause is attested at age 2;0, and then there are several (11) at age 2;2. In questions, the first example is attested at age 2;2, and then two at age 2;3 and eleven at age 2;5. A quick search of three (randomly selected) children in the Manchester corpus (Theakston et al., 2001), Anne, Aran and Joel, reveals that the first attestation of *do-*support in the corpora of these children are at age 1;10.07, 2;0.09 and 1;11.01. In Sunniva’s files, there are no examples of *do-*support at ages 1;6.25 and 1;9.13, and then the first example is attested at age 1;10.01 [*æ don’t* ( = I don’t)]. Then the remaining eleven examples attested before the age of 2;0 are produced at 1;11.22. Thus, with regard to the question of first attestations, Sunniva does not seem that different from the three monolinguals from the Manchester corpus or Susan reported in Miller (1973). However, she does seem to make more extensive use of *do-*support than the three Manchester children at an early stage in development. In the files at ages 1;11.01, 1;11.29 and 2;0.26, which comprise a total of 2677 child utterances, Joel makes use of *do-*support in four instances (0.15%). Anne produces 10 examples among the 4339 utterances she produces at ages 1;10.07, 1;11.06 and 1;11.20 (0.23%). Finally, Aran makes use of *do-*support eight times among his 4139 utterances at ages 1;11.12, 2;0.09 and 2;1.07 (0.19%). In comparison, Sunniva’s 12 examples are uttered in the course of the 1300 utterances she produces at ages 1;6.25, 1;9.13, 1;10.01 and 1;11.22 (0.92%). Thus, even though one from this cannot definitively conclude that she has acquired *do-*support earlier than these monolingual peers, it seems fair to say that she uses *do-*support more than these monolingual children at this early stage of development.

Regarding the question of whether Sunniva’s early use of *do-*support is simply a reflection of her generally being a precocious speaker, there are some indications that this might be the case. In the three early files in Joel’s data (age 1;11.01–2;0.26), his MLU_W_ ranges from 1.299 to 1.846. Anne’s MLU_W_ (aged 1;10.07–2;0.09) is between 1.558 and 2.233, while Aran’s (1;11.12–2;1.07) is 1.299–2.341. In comparison, Sunniva’s four early files (1;6.25–1;11.22) have MLU_W_ between 1.992 and 3.168, which is considerably higher than the three monolinguals, not just in absolute terms, but also in relation to her age. Sunniva’s MLU_W_ is also high in comparison with the other two bilinguals, as illustrated in Table 9.

**Table 9:** Overview of recordings according to MLU for Emma, Emily and Sunniva.

MLU range	Sunniva	Emma	Emily
1.9–2.1	Sunniva (1;6.25)		
	Sunniva (1;9.13)		
	Sunniva (1;10.01)		
2.2–2.4			
2.5–2.7			
2.8–3.0	Sunniva (2;1.16)		Emily (2;3.19)
			Emily (2;3.25)
3.1–3.3	Sunniva (1;11.22)	Emma (2;7.14)	
	Sunniva (2;1.21)		
	Sunniva (2;4.6)		
	Sunniva (2;6.1)		
3.4–3.6		Emma (2;8.17)	
		Emma (2;9.23)	
		Emma (2;10.8)	
3.7–3.9	Sunniva (2;8.0)	Emma (2;9.2)	
4.0–4.2		Emma (2;8.5)	
4.3–4.5			
4.6–5.0			Emily (3;8.18)
			Emily (3;9.25)


#### Summary

The development of residual V2 and especially *do-*support can be shown to follow different paths in the three bilinguals in the current study. Emma exhibits CLI across verb types and across clause types, and thus shows a behaviour compatible with predictions (4a)–(4c). Emily transfers across clause types and makes use of residual V2 and *do-*support in non-subject-initial declaratives, confirming prediction (4b). The data from these two children put together show the validity of predictions (4b) and (4c). Emily’s behaviour demonstrates that residual V2 may be expanded to apply to topicalised structures (4b), thus filling a gap in English in terms of how non-subject-initial structures pattern [cp. (8) and (9) to (12) and (13) in the ‘Verb Placement in Norwegian and English’ section]. Emma’s grammar, however, expands residual V2 into general V2 both in terms of verb types and clause types and thus is in accordance with prediction (4c). However, based on these results, we cannot be sure whether bilingual acquisition in this context could lead to CLI across verb types only (4a), resulting in a grammar where all verb types, including lexical verbs, may precede the negation in negative declaratives and subjects in questions, but not in topicalisations. Sunniva, on the other hand, does not exhibit any kind of transfer and is very precocious in her use of *do-*support. However, as she also appears to have a higher MLU_W_ in relation to her age than both the three monolinguals we have compared her to and the other bilinguals in the current study, this is most likely not due to accelerated development as a result of CLI. Consequently, we cannot draw any conclusions regarding prediction (4d). However, these results together confirm prediction (5), as the simultaneous exposure to the V2 language Norwegian and residual V2 in English seems to result in different developmental paths for the acquisition of residual V2 and particularly *do-*support. Thus, it appears that different parsers may interpret the input differently in bilingual situations such as these. The question is what exactly might cause this to happen, specifically whether language dominance can explain the observed differences.

### Dominance as an Explanation for the Different Developmental Paths

The final research question addresses to what extent the differences between the three children can be explained with reference to language dominance, the underlying assumption being that CLI is more likely to occur from the dominant to the weaker language (for studies partially supporting this view, see, e.g. Bernardini and Schlyter, 2004; Nicoladis, 2006, 2012; Argyri and Sorace, 2007; Silva-Corvalán, 2014). As we have seen, Emma and Emily are affected by CLI, and consequently, we might expect Norwegian to be the dominant, or at least the stronger language for these girls. Sunniva, on the other hand, does not seem to be affected by CLI in the English structures under scrutiny, suggesting that her English is stronger, maybe even the dominant language for her. However, CLI is manifested in different ways in Emma and Emily, and another question pertains to whether these differences also can be explained by language dominance. Dominance has been argued to be an inherently gradient dimension (cf., e.g. Grosjean, 1982; Kupisch and Bernardini, 2007; Luk and Bialystok, 2013; Birdsong, 2015), and as such it should be possible for one of the girls to be more dominant in Norwegian than the other. If this were the case, the (inconsistent) whole-sale transfer of V2 observed in Emma’s data would be indicative of a stronger Norwegian dominance for her compared to Emily, who displays a very specific transfer of residual V2 to topicalisations. However, recall from the discussion in the ‘Materials and Methods’ section, that we only have English data for Emily, so any comparison between the two will have to be made on the basis of English only.

The notion of dominance is ubiquitous in much of the literature on bilingualism, irrespective of whether the object of study is simultaneous or sequential bilingual child language acquisition, adult L2, or adult heritage speakers. In any bilingual situation, the question of which language is the stronger one tends to be important and relevant. Despite this, a wide variety of measures have been used to determine language dominance, and there is consequently no generally agreed upon indicator available. The most frequently used measures relate to the (relative) level of proficiency in the two languages and/or the (relative) exposure to and use of the two languages (see, e.g. Kupisch and Bernardini, 2007; Silva-Corvalán and Treffers-Daller, 2015; Unsworth, 2015). Montrul (2015) includes all these three as different dimensions of dominance: the speaker’s comparative proficiency, input situation and opportunity to use the languages. However, a recent paper (Lloyd-Smith et al., unpublished) introduces the experience-to-outcomes hypothesis to explain the wide range of variation usually observed in adult or adolescent heritage speakers, proposing that it is the sum of the speaker’s experience in the heritage language that determines how proficient s/he becomes. This makes level of proficiency the result of the amount and quality of input and opportunities to use the language, rather than an interacting factor. In the end, the definition of dominance and the means used to measure it is to some extent dependent on the population investigated. For example, in adult or adolescent heritage speakers, amount of exposure and use may easily be operationalised as majority language, as the speakers clearly will have had more exposure to and opportunity to use this than the heritage language (see, e.g. Kupisch and van de Weijer, 2015). With young bilinguals, the situation is clearly different. Even though the three children in the current study will most likely end up with having had more exposure to Norwegian, this is not necessarily the case early on in the development, especially given their linguistic situations with English as their home language. Equivalently, even though they probably will end up more proficient in the majority language, this might not be the case at an early stage. Thus, in the current study, we discuss both language use and proficiency to determine to what extent dominance can explain the different behaviours of the three children. However, as the information available regarding language exposure and use is more limited, the main focus will be on proficiency.

There are no obvious objective measures available with respect to language exposure and use in the three small corpora. The families were not asked to fill in any questionnaires about language use, and the recordings were made so long ago that there is no reliable way of obtaining this information from the parents today. However, the language situations are very similar for the three girls. Recall from ‘Materials and Methods’ section that they all attended Norwegian nursery from approximately the age of one, and the families are strongly integrated with the community at large, thus ensuring exposure to and use of Norwegian from early on. With respect to the home language situation, all three girls grew up with one parent who is a native speaker of English, and one who is a native speaker of Norwegian, and they all have English as their home language. The non-native parents are highly proficient in English and do not make the kinds of mistakes that we have observed in two of the children. Indeed, these kinds of errors are lost early in the L2 acquisition of English by Norwegian learners (Westergaard, 2003). Moreover, two of the girls, Sunniva and Emily, even grew up in the same family, making it less likely that huge variation in exposure to and use of English has caused the differences between them. If anything, Emily would have benefitted from the extra input from her older brother and sister. Furthermore, when she was born, the English grandparents were retired, which made it possible for them to visit their grandchildren more and for longer periods than when the older siblings were small. These facts together suggest that all the three children have a relatively balanced input situation, possibly slightly dominated by English at the earliest stage.

However, one possible explanation for the differences between the sisters might simply be that the data that we have available do not capture the period when Sunniva exhibits the same behaviour as Emily. Recall that Emily’s non-target-like topicalised structures occur in the later files (at 3;8.18 and 3;9.25), while there are no non-subject-initial declaratives in Emily’s early files (2;3.19 and 2;3.25).^7^ Sunniva was recorded between the age of 1;6.25 and 2;8.0, and in this period, she only produces two (target-like) topicalised structures. Thus, Sunniva potentially may have gone through a period *after* data collection finished when she exhibited the same behaviour as Emily.

When proficiency has been used as an indicator of dominance, many different types of measures may be used to determine the balance between the two languages. MLU (sometimes with additional measures and/or specific implementations) is frequently been used in corpus studies (see, e.g. Genesee et al., 1995; Yip and Matthews, 2000, 2006; Bernardini and Schlyter, 2004; Kupisch and Bernardini, 2007; Hager and Müller, 2015). A general problem with the use of MLU to compare proficiency in the two languages of a bilingual is that languages differ greatly with regard to morphological complexity (Döpke, 1998; Yip and Matthews, 2006). This has been pointed out to be problematic for languages such as Italian and Swedish (Bernardini and Schlyter, 2004), as a comparison in terms of MLU_W_ underdetermines the score in Swedish compared to Italian because the Swedish definite article is suffixal, while the Italian one is a free morpheme. Consequently, only the latter would be included in an MLU_W_ count.^8^ Note that the same difference applies between the two languages investigated here, Norwegian and English (*bil-en* versus *the* car). From this perspective, a higher MLU_W_ is expected in English, all other things being equal. Apart from this specific fact, the two languages are relatively similar with regard to morphological complexity and the realisation of various functional elements as free or bound.

**FIGURE 1:**
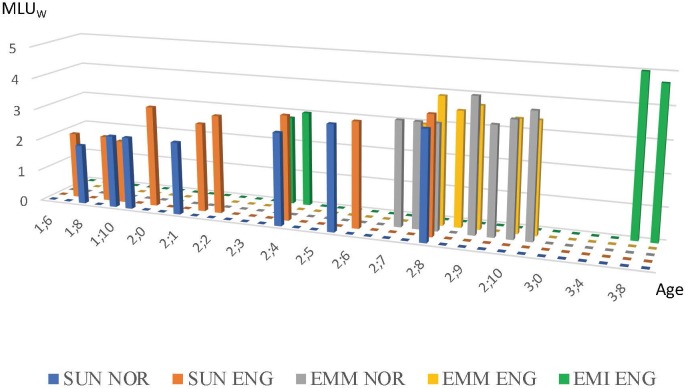
Overview of MLU_W_ in both languages according to age for Emma, Emily and Sunniva.

An overview of the three children’s MLU_W_ is provided in Figure 1. A visual comparison between Sunniva’s MLU_W_ in English and Norwegian suggests that it is higher in English, especially between the age of 2;0 and 2;6. Emma’s MLU_W_ appears to be more similar in the two languages, with English peaking in one file and Norwegian in another. This impression is confirmed if we work out the average MLU for all the files in the each of the languages for Emma and Sunniva [inspired by Arencibia Guerra, 2008’s measure of mean MLU difference (MMLUD), reported in Hager and Müller, 2015]. Emma’s average MLU_W_ is exactly the same in the two languages (3.562), while Sunniva’s average for English is 2.833 and for Norwegian 2.648. On this measure, both Emma and Sunniva would be classified as ‘strongly balanced’ according to Arencibia Guerra’s (2008) criteria (there is less than a 0.29 difference between the languages). Recall, however, that MLU_W_ may to underdetermine the score for Norwegian compared to English because of the different status of definite article in the two languages. Nevertheless, with respect to MLU_W_, both Emma and Sunniva are very balanced.

Another possible indicator of dominance is language mixing, as the direction of mixing often is claimed to occur from the stronger to the weaker language (cf. Genesee et al., 1995; Bernardini and Schlyter, 2004; Lanza, 2004; Kupisch, 2007; Kupisch and Bernardini, 2007, but see Anderssen and Bentzen, 2013). We only consider non-syntactic mixing in order to avoid that the instances of non-target-like verb movement investigated in the current study affects the measure of language dominance. Given previous studies, it is likely that the proportion of mixed utterances in the files of the three children (in both languages for Sunniva and Emma and English only for Emily) might give us an indication of which language the children is most proficient in. The language with the highest proportion of utterances with mixing should be the weaker, non-dominant one.

Correlating language mixing as a measure of language dominance and balance, we would expect both Emily and Emma to have a higher proportion of mixing in their English than Sunniva, possibly also with Emma mixing somewhat more than Emily (on the assumption that dominance can be gradient). However, as we can see from Table 10, this is not the case. In fact, Emma is the one with the lowest proportion of mixing in both languages, with 2.7% for English and 1.1% in Norwegian. Sunniva, who is the most target-like of the three children, mixes 8.2% in English and 4.9% in Norwegian. Finally, Emily, who we only have English files for, displays 13.4% mixes. Examples of the different kinds of mixing are provided in (55)–(57).

(55) It wasn’t *ordentlig* on *egentlig*. (Emily)
        TARGET: It wasn’t *properly* on *really*.
(56) *Nå skal* dolly *sove*. (Sunniva)
        TARGET: *Now* the dolly *is going to sleep*.
(57) I falled on *en tå.* (Emma)
        TARGET: I fell on *a toe*.

**Table 10:** Language mixing with words, phrases and sentences in Emma, Emily and Sunniva.

Child/language	Total no. utterances	Word mixing	Phrasal mixing	Sentence mixing	Total mixing
Emma/ENG	1831	32 (1.7%)	8 (0.4%)	10 (0.5%)	50 (2.7%)
Emma/NOR	2222	14 (0.6%)	4 (0.2%)	6 (0.3%)	24 (1.1%)
Emily/ENG	1495	145 (9.7%)	35 (2.3%)	20 (1.3%)	200 (13.4%)
Emily/NOR	Not applicable				
Sunniva/ENG	2512	167 (6.6%)	19 (0.8%)	20 (0.8%)	206 (8.2%)
Sunniva/NOR	2890	123 (4.3%)	12 (0.4%)	6 (0.2%)	141 (4.9%)


These results thus reveal two things: (i) both Sunniva and Emma, the two children we have both English and Norwegian files from, mix more in their English than in the majority language and (ii) the proportion of mixing in the children’s English does not seem to be correlated with the extent to which they behave target-like with residual V2 and *do-*support. Emma, the child who is the most influenced by Norwegian, mixes less than Sunniva, who appears to be the most target-like with respect to English verb placement. However, another factor also pertains to who the interlocutor is in the different files. In Emma’s English files, she is mostly with her American mother, while in her Norwegian files, she is playing with an investigator whom she believes does not speak English. Sunniva’s English files mostly include her Norwegian (but English-speaking) mother, while the Norwegian files are recorded with a Norwegian investigator. Note, however, that the mother is almost always present. Emily falls in between Sunniva and Emma in terms of acquisition of SAI and *do-*support but mixes the most of all of them. Emily’s (English) files mainly include her Norwegian (but English-speaking) mother. One relevant question is why Emily mixes more than the other two. A factor contributing to this might be that Emily’s older brother code-switches quite extensively, and in the context of a family where all the members are very fluent in both languages, code-switching is thus a natural communicative strategy also for Emily.

Further arguments against an explanation in terms of language dominance are provided in other studies involving Sunniva and Emma. Anderssen and Bentzen (2013) investigate modified definite DPs in Emma’s Norwegian, finding overgeneralisation from English into Norwegian with respect to definiteness. They explain this behaviour with reference to simplicity, as these structures involve so-called double definiteness in Norwegian. Importantly, this shows that CLI may also go from English into Norwegian in Emma’s languages. Another study investigates the acquisition of gender in two monolingual Norwegian children as well as Emma and Sunniva (Rodina and Westergaard, 2013). With regard to this phenomenon, Sunniva and one of the monolingual children pattern together and are very target-like. Emma patterns with the other monolingual child, and both are non-target-like. This indicates that Sunniva is most likely more advanced than Emma in Norwegian as well.

To sum up, it appears that all the measures of dominance available to us indicate that Emma and Sunniva are fairly balanced bilinguals. Both of them have very similar MLU_W_s in English and Norwegian, and they mix more Norwegian into their English than the other way around. For Emily, we do not have access to Norwegian data, and thus cannot compare her English and her Norwegian competence. However, overall, she is not particularly delayed in her acquisition of English, which one might expect if Norwegian was strongly dominant. Furthermore, the rate of mixing does not seem to reflect the extent to which the three girls are target-like in their behaviour. Finally, the fact that English is their home language combined with frequent exposure to the community language supports the impression of three bilinguals who are very balanced. This also means that prediction (6) is not confirmed. The differences between the children cannot be explained with reference to language dominance. Rather, it seems that the three children (at least Sunniva and Emma) behave differently despite being relatively similar with regard to language balance. In the next section, we explore to what extent the different behaviours can be accounted for with reference to the two linguistic systems.

### Structural Ambiguity as an Explanation for CLI

So far, we have seen that the three bilingual children investigated in the current study behave very target-like when it comes to auxiliary placement in negative structures and questions. However, in contexts requiring *do-*support, the three children diverge. While Emily and Sunniva are very target-like also in these contexts, Emma produces a high proportion of non-target-like utterances. Recall from ‘Results’ section that unlike the other two, Emma employs verb movement of lexical verbs across the negation in negative structures and across the subject in *yes/no-*questions, suggesting that she has overgeneralised residual V2 to apply across verb types. Furthermore, she also allows both SAI and verb movement of lexical verbs in non-subject-initial declaratives, suggesting that she has overgeneralised V2 to apply across clause types as well, thus confirming predictions (4b) and (4c). For Emily, we have seen that even though her behaviour is very target-like in structures involving residual V2, she overgeneralises auxiliary movement to topicalised structures, and as a result, the majority of her non-subject-initial declaratives involve non-target-like SAI or *do-*support. We have further seen that language dominance, at least as it can be measured with these data, cannot explain the differences between the children. Nevertheless, we observe that the parsers of the three children somehow interpret the data differently.

Recall from ‘Residual Verb Second and *Do*-Support in English’ section that even though English does not make use of SAI or verb movement in non-subject-initial declaratives, adverbials such as *here* and *there* in initial position may cooccur with V2 [cf. (14) and (15)]. One possible explanation for Emily and Emma’s non-target-like behaviour with topicalisations is that they have been exposed to a large number of these structures in the input, with a high proportion involving DP subjects, causing them to overgeneralise V2 into topicalisations in English. If this is the case, we would expect Emma and Emily to have had more exposure to and make more use of such structures than Sunniva. However, as illustrated by Table 11, both Emma and Emily appear to have considerably fewer of these topics in their input than Sunniva (12 and 52 versus 133). Also, while there is a slight majority of these structures with DP subjects, and hence V2, in the production of the adult speakers, the distribution is quite even. Similarly, the extent to which the children topicalise *here/there* is not completely in line with what the adult speakers in the same corpus do. Notably, Emma makes use of these topics more than her mother (19 versus 12), while Emily is the one with the highest number of these structures (32, compared to 52 by her mother and sister). The distribution of ±V2 is also quite similar to that of the adults, but slightly more skewed towards V2. Emma is the one with the clearest preference for V2 in these structures (89.5%), but these are all target-like. It thus seems that the frequency of these structures in the input cannot account for the variation among the children (even though these corpora clearly are very limited).

**Table 11:** The use of + /-SAI in topicalisations with *here/there* adult and child speakers in the corpora.

Speaker	DP	Pronoun	Total	Total
	with SAI	without SAI	*here/there*	adult input
EMMA mother	7 (58.3%)	5 (41.7%)	12	12
EMMA	17 (89.5%)	2 (10.5%)	19	
EMILY mother	24 (50%)	24 (50%)	50	52
EMILY sister	0	2 (100%)	2	
EMILY	19 (59.4%)	13 (40.6%)	32	
SUNNIVA mother	57 (55.3%)	46 (44.7%)	103	133
SUNNIVA father	15 (50%)	15 (50%)	30	
SUNNIVA	12 (60%)	8 (40%)	20	


Interestingly, however, an investigation into the children’s behaviour with DP versus pronominal subjects in non-subject-initial declaratives suggests that the two children who overgeneralise residual V2 are indeed influenced by the word order variation found with *here* and *there*. As demonstrated in Table 12, there is a strong tendency for both Emma and Emily to make use of V2 in exactly those cases where the subject is a DP, and not only when *here/there* are topicalised. The first two columns in the table show the children’s use of V2 (+V2) with DP subjects and V3 (-V2) with pronouns when the topic is *here* or *there*. As the table reveals, the children follow this pattern completely. Then the next six columns show the same distribution (+V2/DP subject and -V2/pronominal subject) with other topics (e.g. *now, then* or *maybe*) and with lexical verbs, auxiliaries and copula *be* and *do*, respectively. Note that in these columns any structure that is +V2 is ungrammatical, but the closer the percentage in each of these columns is to 100%, the more similar the child’s behaviour with topics in general is to *here/there*. As we can see, Emily consistently has V2 with DP subjects, except with lexical verbs, which she does not allow in the V2 position. With pronominal subjects, her behaviour is more variable, but clearly a substantial amount of her pronominal subjects also occurs with V2 (9/28, if we disregard lexical verbs), thus going against the pattern. Emma exhibits a high preference for both V2 with DP subjects (87.5%, but only auxiliaries are attested with DP subjects) and for V3 with pronominal ones (78.6, 77.4 and 100%). What is surprising is that it is the two children who appear to have been exposed to these structures the least who have adopted the word order pattern with V2. One possible explanation for this might be that the children need exposure to a certain number of examples to realise that structures such as non-subject-initial declaratives with *here/there* actually represent an exception. In the absence of sufficient exposure, the parser makes an overgeneralisation based on the available data, which for Norwegian/English bilinguals also will include data with massive indications of V2. Moreover, V-to-T movement of auxiliaries in English causes negative declaratives to superficially look similar to Norwegian constructions involving V2. The same is true for positive declaratives with adverbials such as *often* and *always* when they include an auxiliary. In the absence of auxiliaries, however, the similarity breaks down. This division between auxiliaries and lexical verbs makes the English system more ambiguous than the Norwegian one, leaving it open to several possible interpretations.^9^ According to Henry and Tangney (1999: 139), ‘language acquisition involves tension between the drive to create a maximally simple grammar in Universal Grammar (UG) terms and the need to adopt a grammar that covers the input data’; there is little doubt that the simultaneous exposure to English and Norwegian causes Norwegian to influence English residual V2. On the assumption that Henry and Tangney are correct, it is no surprise that CLI goes in this direction, as Norwegian V2, which is consistent across verb types and clause types, can be described as much more coherent than English residual V2. English is less consistent, with V2 only applying to certain structures (questions, negation and some topicalised structures) and specific verbs (auxiliaries, copula and in many cases *do*).

**Table 12:** Subject typesand verb placement in topicalisations with *here/there* (for Emma, Emily and Sunniva) compared to other topics (for Emma and Emily only), divided into verb types.

Speaker	Here/there (%)		Other topics (%)		Other topics (%)		Other topics (%)	
			lexical verbs		Aux and *be*		*do*	
				
	+V2/DP	-V2/Pr	+V2/DP	-V2/Pr	+V2/DP	-V2/Pr	+V2/DP	-V2/Pr
Emma	17/17	2/2	0/0	22/28	7/8	24/31	0/0	1/1
	(100%)	(100%)	(0%)	(78.6%)	(87.5%)	(77.4%)	(0%)	(100%)
Emily	19/19	13/13	0/9	7/7	10/11	6/22	21/21	3/6
	(100%)	(100%)	(0%)	(100%)	(90.9%)	(27.3%)	(100%)	(50%)
Sunniva	12/12	8/8	–	1/1	–	1/1	–	–
	(100%)	(100%)		(100%)		(100%)		


## Conclusion

This paper investigates the acquisition of residual V2 in three Norwegian-English bilinguals. We find that the three girls exhibit three different patterns with regard to the relevant constructions, despite the fact that they grew up in comparable language situations. We argue that the non-target-like behaviour with respect to verb placement and *do-*support is caused by CLI from Norwegian. Furthermore, we have discussed various possible explanations for the differences between the three children’s acquisition of verb placement in English. It is not obvious that the differences between the children can be explained with reference to language dominance, nor can they be explained in terms of frequency of exposure to non-subject-initial structures exhibiting optional V2. We have suggested that the observed CLI can be accounted for by the ambiguity in the English system, which leaves the data open to several possible interpretations when English is acquired in contact with the consistent V2 system in Norwegian. It thus seems that the children’s parsers may interpret the input differently. Importantly, this means that the differences between the children are qualitative rather than quantitative. Furthermore, for Emily, we also know that she was able to ‘recover’ from this grammar, and we assume the same is true for Emma (who is an adult now), suggesting that this kind of recovery has to be possible and needs to be accounted for in developing grammars. We leave to future research the question of how such recovery from a non-target-like grammar is possible.

## Ethics Statement

This research is based on child language corpora collected in a period from 1999 to 2012. Parents were thoroughly informed about the data collection and the purpose of this, and signed consent forms on behalf of themselves and the children we collected data from. At the point in time when these data were collected, there were no requirements of approval from an ethics committee in Norway, and thus, such approval has not been obtained specifically for the collection of these data. However, approval has been obtained from NSD – Norwegian Centre for Research Data for the overall research project MiMS (Micro-Variation in Multilingual Acquisition and Attrition Situations) which the current investigation is a part of.

## Author Contributions

All authors listed have made substantial, direct and intellectual contribution to the work, and approved it for publication.

## Conflict of Interest Statement

The authors declare that the research was conducted in the absence of any commercial or financial relationships that could be construed as a potential conflict of interest. The reviewer CP and handling Editor declared their shared affiliation.

## Footnotes

1 In some dialects of Norwegian, including the dialect acquired by the three children in this study, inversion of the subject and the finite verbal element in *wh*-questions depends on type of *wh*-word, verb type, subject type and information structure. We will not specifically address this issue in the current paper, and we refer the reader to Westergaard (2009) for a discussion of this phenomenon.

2 This also applies to other types of topics. As shown in (i) and (ii), negative and restrictive elements typically trigger SAI:

(i) Only then *did* he realise…
(ii) Never before *have* I seen a more beautiful….

Furthermore, verbs of reporting frequently invert when they follow direct speech, as in (iii). Note, however, that such verbs display a variation similar to that of *here* and *there* with regard to the placement of full DP and pronominal subjects [(iii)] versus [(iv)]:

(iii) ‘Where is he now?’, *said* Mary.
(iv) ‘Where is he now?’, she *said*.

Finally, unaccusative verbs such as *arrive*, *sit* or *depart* may also precede the subject in topicalised structures, especially but not exclusively with locative elements [see (v) and (vi)]:

(v) Then *arrived* the big stars.
(vi) In the corner *sat* a mysterious stranger.

3 All percentages provided in the discussion of Thornton and Rombough’s results are our own calculations.

4 We obtained written and informed consent from the children’s parents on behalf of both their children’s and their own participation in the corpus collection. At the time when these corpora were collected, there were no national requirements for approval of such data collection in Norway. However, the subsequent project which the current project is a part of, *Micro-variation in Multilingual Acquisition (MiMS)*, has gained approval from the *Norwegian Centre for Research Data (NSD* – http://www.nsd.uib.no/nsd/english/index.html).

5 Given that the Tromsø dialect allows non-V2 in certain *wh*-questions, see footnote 1, structures such as (33) could be CLI from Norwegian. However, it is also possible that these are just examples of a type of behaviour that is sometimes observed in monolingual English children.

6 Note also that *all* topicalised elements were searched out, but very few these were arguments. The vast majority involved temporal and locative elements.

7 There is one exception to this and that involves topicalised structures with *here* or *there* as topics, since they exhibit variable word order (see ‘Residual Verb Second and *Do*-Support in English’ section). These are also not included in Sunniva’s data on non-subject-initial declaratives.

8 As pointed out by a reviewer, Italian is a pro-drop language while Swedish is not. Thus, with respect to this feature, MLU_W_ should be higher in Swedish than Italian.

9 See also Biberauer and Roberts (2017) for similar reasoning concerning how parameter setting may change diachronically.
